# Acute dystonic reactions in a lady presenting with repetitive involuntary muscle twitching: a case report

**DOI:** 10.1186/1757-1626-2-186

**Published:** 2009-11-09

**Authors:** Moe Thaw Oo

**Affiliations:** 1 Specialist Registrar, Geriatric and Stroke Medicine, Royal Shrewsbury Hospital, Shrewsbury, SY3 8XQ UK

## Abstract

A 56-year-old lady was admitted with complaint of involuntary muscle twitching around the eyes, face and neck for two days. She had a history of low grade non-hodgkin lymphoma with completion of the first cycle of chemotherapy. Her medication on presenting consisted of Ondansetron 8 mg two times a day and Metoclopramide 10 mg three times a day (TDS). She started taking these medications 24 hours before having the above symptoms. She was clinically diagnosed with acute dystonic reactions and was also secondarily treated with anti-emetic medications. She was given IV procyclidine 10 mg stat followed by per oral (PO) procyclidine 2.5 mg TDS. Within an hour of administering IV procyclidine her symptoms began to gradually settle down.

Acute dystonic reactions are not a very rare clinical presentation in the daily practice. The above case is a good example for the clinicians dealing with acute medical admissions.

## Case presentation

A 56 year old lady was admitted with complaint of involuntary muscle twitching around eyes, face and neck. She presented with acute onset of repetitive muscle twitch and nausea within 48 hours after completing the first cycle of chemotherapy for low grade non-hodgkin lymphoma. She was taking Ondansetron 8 mg BD and Metoclopramide 10 mg TDS. She started taking these medications 24 hours prior to appearance of above symptoms.

On admission she was anxious and worried for having stroke or seizures. There was no history suggestive of similar symptoms in past. No other significant medical illness was also noted.

Physical examination revealed transient non-specific facial muscles twitchings. Rest of the general and systemic examination was unremarkable. All baseline blood tests results (Full Blood Count, Urea and Electrolytes, Liver Function Tests), CXR and ECG findings were normal. She was clinically diagnosed as acute dystonic reactions secondary to anti-emetic medications. All the medications were discontinued and she was treated with IV procyclidine 10 mg stat followed by PO procyclidine 2.5 mg TDS. She was also commenced on PO domperidone 10 mg PRN/QDS for her nauseous feelings. Within an hour of receiving IV procyclidine her symptoms gradually improved and disappeared. She was discharged with complete recovery within 24 hour hospital admission.

## Discussion

Acute dystonic reactions have different manifestations (Table 1)). These reactions are usually occured as a side effect of neuroleptic and anti-emetic medications. The clinical spectrum is poorly understood and frequently led to misdiagnosis.

Causes or triggering factors include: neuroleptics, amantadine, benzodiazepines, carbamazepine, chloroquine, cisplatin, diazoxide, influenza vaccine, levodopa, lithium, metoclopramide, nifedipine, pemoline, phencyclidine, reserpine, tricyclics, postencephalitic Parkinson's, Tourette's syndrome, multiple sclerosis, neurosyphilis, head trauma, bilateral thalamic infarction, lesions of the fourth ventricle, cystic glioma of the 3^rd ^ventricle, herpes encephalitis, juvenile Parkinson's. It is often not realized that in addition to the acute presentation, it can develop as a recurrent syndrome, triggered by stress, and exposure to the above drugs [1].

**Table 1:** Manifestations of acute dystonia [2]

Occulogyric crisis (OGC)	Spasm of extraorbital muscles, facial muscles, involvement of upward and outward deviation of eyes
Torticollis	Head held turn to one side

Opisthotonus	Painful forced extension of the neck

Macroglossia	Protrusion of tongue and seemed to be swollen

Buccolingual crisis	Trismus, Dysarthria and Grimacing

Laryngospasm	Uncommon but frightening

Spasticity	Trunk muscles and less commonly limbs can be affected

Transient ischaemic attacks (TIA), focal seizures or other involuntary muscle tics and spasms should be considered in differential diagnosis. The detailed history taking and thorough neurology examination help in reaching the correct diagnosis. Imaging studies of brain should be conducted to exclude other intracranial pathology and structural abnormalities leading to the above mentioned manifestations, especially if these symptoms persist.

Treatment in the acute phase of dystonic reactions involves reassurance and treatment with Procyclidine and/or Benztropine and/or Diazepam or lorazepam. Maintenance therapy with oral forms of the above medications or amantadine are indicated in more chronic recurrent cases [1].

## Conclusion

It is a distressing complication of antiemtic and antipsychotic drugs. In the acute clinical settings these unpleasant symptoms can make the patients anxious and the diagnosis can be confused with other acute medical conditions such as transient ischaemic attacks. The prompt therapeutic action is essential in its management. It is not a very rare case in the daily practice. The above case is a good example for physicians dealing with acute medical admissions during on-calls.

## List of abbreviations

BD: Two times a day; CXR: Chest X'Ray; ECG: Electrocardiogram; IV: Intravenous injection; PO: Per oral; PRN: As required; QDS: Four times a day; TDS: Three times a day.

## Competing interests

The author declares that they have no competing interests.

## Authors' contributions

MTO is the only one and main author. No other author was contributed in this report.

## Consent

The valid informed consent was taken from the patient for this case report and publication.
